# Chiropractor perspectives on the use of patient reported outcome measures: a survey of Veterans Health Administration chiropractors

**DOI:** 10.1186/s12998-026-00631-5

**Published:** 2026-03-18

**Authors:** Brian C. Coleman, Cynthia A. Brandt, Edward R. Melnick, Todd Kawecki, Alicia Heapy, Anthony J. Lisi

**Affiliations:** 1 Pain Research, Informatics, Multimorbidities, and Education (PRIME) Center, VA Connecticut Healthcare System, 11-ACSL-G, 950 Campbell Avenue, CT 06516 West Haven, USA; 2 Yale School of Medicine, Yale University, New Haven, CT USA

**Keywords:** Veterans health, Patient reported outcome measures, Quality of health care, Chiropractic

## Abstract

**Background:**

Patient reported outcome measure (PROM) use is a marker of high-quality chiropractic care. Limited information exists regarding use of PROMs by United States chiropractors, especially within integrated health systems like the Veterans Health Administration (VHA) that prioritize high-quality, data-driven performance monitoring. This study aimed to understand VHA chiropractor perspectives on PROM use, including specific measures, administration characteristics (initial and follow-up timing), workflow, and perceived utility.

**Methods:**

A national electronic survey of VHA chiropractors was conducted using REDCap from May 7 to 28, 2024. Survey items covered frequency of use by region of chief concern, administration during initial versus follow-up visits, perceived usefulness for serial assessment, and perceived patient and clinician benefit or burden. Respondents also listed frequently used PROMs and provided details on administration workflow. Descriptive statistics were summarized. The relationship between self-reported PROM use at initial and follow-up visits was compared using the Wilcoxon signed-rank test (α = 0.05). Self-reported PROM use frequency across chief concerns (low back pain, neck pain, and all other chief complaints) was compared using a Kruskal-Wallis H Test (α = 0.05) and Bonferroni-corrected Dunn’s post-hoc tests.

**Results:**

The survey achieved a response rate of 51.8% (189/365). The PROMs most frequently listed assessed disability, followed by pain intensity and interference. PROM administration was most often completed by the chiropractor (76.7%) using paper forms collected at the point of care (51.7%). Respondents reported a greater (*r* = 0.640, *p *< 0.001) percentage of patients over the past 12 months where they used PROMs at initial visits (median = 95.0%) compared to follow-up visits (median = 74.5%). A significant difference in PROM frequency was observed across chief complaint categories (χ² = 41.814, df = 2, *p *< 0.001) for both low back pain (*r* = 0.258, *p *< 0.001) and neck pain (*r* = 0.203, *p *< 0.001) compared to all other chief complaints. PROMs were overwhelmingly viewed as highly beneficial for individual patient management despite any perceived administrative burden.

**Conclusions:**

We identified key themes related to VHA chiropractor perspectives on PROMs. VHA chiropractors show a commitment to PROM use, especially for spinal pain presentations. Existing workflows often rely on manual, paper-based, provider-driven methods. Addressing barriers to meaningful use is critical to optimally implement PROMs in VHA chiropractic practice.

**Supplementary Information:**

The online version contains supplementary material available at 10.1186/s12998-026-00631-5.

## Introduction

Patient-reported outcome measures (PROMs) are essential tools for capturing patient experience, assessing perceptions of their health status, and monitoring treatment effectiveness [1]. PROMs provide patient-centered data that complement objective clinical findings and support a holistic view of patient health. The clinical utility of PROMs is broad: their use may facilitate shared decision-making, improve communication between patients and providers, and contribute to improved patient outcomes [2, 3]. Further, their use as a component of data-driven, measurement-based care is useful for quality improvement, population health monitoring, and successful delivery within a value-based care model [4, 5].

Despite these established benefits, the successful, large-scale implementation of PROMs in routine clinical practice has been challenging. Barriers frequently cited include the administrative burden of collection, difficulty integrating measures into existing clinical workflows, ensuring the selection of appropriate validated tools, and variation in perceived clinical utility of and providers’ skepticism about their results [3, 6]. Musculoskeletal pain care best practices recommend progress evaluation using PROMs [7, 8], but without objective guidance on the optimal timing of initial and follow-up assessment or the frequency of repeat measure collection (i.e., repetition after a specific number of visits or period of time). The absence of a clear “gold-standard” recommendation likely complicates PROM uptake in clinical practice by adding unnecessary cognitive load to clinicians [9], who must either recall any measure-specific administration recommendations or independently devise their own schedules for serial measurement. Understanding these facilitators, barriers, and determinants of effective implementation is paramount for developing implementation strategies.

PROM use is particularly important in the management of chronic and complex musculoskeletal conditions [7], including as part of chiropractic care [10, 11]. Chiropractic care has become increasingly integrated within the U.S. Veterans Health Administration (VHA) as part of a comprehensive, multidisciplinary approach to musculoskeletal conditions for Veterans [12, 13]. The VHA system emphasizes the importance of high-quality, data-driven care with performance monitoring [14]. There is added importance as the chiropractic program is expected to continue to grow in the coming years [15]. Previous empirical analyses using natural language processing on a national collection of VHA chiropractic care electronic health record (EHR) data confirmed that documentation related to PROMs is present in clinical notes at below target levels (17% of all notes and 36% of unique patients) [16]. However, these data provide no context for the under-expected use of PROMS. They do not clarify how VHA chiropractors choose, administer, and interpret these measures in real-world practice, nor do they capture the perspectives of the providers themselves.

This highlights a critical literature and implementation gap: while the use of PROMs is widely accepted as a best practice, the specific implementation strategies, choice of measures, and perceived utility can vary substantially. Studies of Australian chiropractors on PROM implementation have described barriers and facilitators to clinical adoption [17], clinician perceptions on value [18], and the effectiveness of a targeted education implementation strategy [19]. However, there is limited understanding of how PROMs are used in practice by chiropractors in the United States, particularly those working within the unique and complex environment of an integrated health system like the VHA. This study aimed to understand VHA chiropractor perspectives on the use of PROMs in clinical practice, including PROMs used, administration characteristics (including distributions at initial and follow-up visit time-points), workflow characteristics, and perceived utility. Addressing this gap is critical to optimizing clinical workflows and maximizing the value of chiropractic services for Veteran patients.

## Methods

### Study design and participant recruitment

A cross-sectional national electronic survey of VHA chiropractors was conducted using administration methods consistent with a prior national VHA chiropractic field survey [20]. All VHA chiropractors privileged at VHA facilities at the time of the survey were identified using VHA Chiropractic Program Office internal tracking data. Eligible participants included full- or part-time privileged chiropractors (including employees and contractors). VHA trainees (e.g., student clerks, residents, fellows) were ineligible to participate. Study team investigators were excluded. The survey was conducted as part of an exempt study approved by the Institutional Review Boards of the VA Connecticut Healthcare System Research & Development Committee (#1690344) and the Yale University Institutional Review Board (#2000032830).

### Survey development

The electronic survey was developed to understand VHA chiropractor stakeholder perspectives and priorities across three related domains: (1) the use of PROMs in VHA chiropractic care, (2) the use of clinical decision support (CDS) by VHA chiropractors, and (3) perceptions of acceptance, usability, and appropriateness of CDS during chiropractic care to promote PROM use. This study describes the perspectives of VHA chiropractors on the use of PROMs in VHA chiropractic care. Data regarding the remaining domains (CDS use and perceptions of technology implementation) are reserved for a separate analysis focused specifically on technology integration and decision support.

The survey instrument structure and content were finalized through an iterative pilot-testing and revision approach. An initial version was pilot-tested with a sample of five current VHA chiropractors, with revisions incorporated for enhancing clarity in the final version. The final instrument included 62 total items and is available on the Open Science Framework. Respondent demographics were collected using 10 items. There were 16 items describing current use of PROMs. For the purposes of the survey, PROMs were explicitly defined as “standardized, validated questionnaires completed by patients to identify and quantify their perceptions of their health status”. PROM use characteristics included frequency of use by region of chief concern, frequency of use during initial or follow-up visits, perceived usefulness of PROMs for serial assessment, and perceived benefit or burden for the patient and clinician. Respondents were also asked to list PROMs that they frequently use in their practice for low back pain, neck pain, and any other chief concern. PROM administration methods were captured using 2 items. The remaining 34 items related to the general CDS use and CDS to promote PROM use domains.

### Data collection

Research Electronic Data Capture (REDCap) was used to curate the survey instruments, collect responses, and manage response data [21]. Potential survey respondents were invited by email to participate with a unique link distributed through the REDCap system at the survey launch. The survey was open for responses for three weeks (from May 7, 2024 to May 28, 2024). Reminder emails were distributed at 7-, 14-, and 20-days following survey launch to all potential respondents who had not yet completed the survey or opted out of future contact. Survey responses were collected anonymously, and all participation was voluntary. Voluntary demographic items could be skipped or marked as “Prefer not to answer”. Responses were exported from REDCap for statistical analysis in RStudio (Boston, MA) using R version 4.4.0 (R Core Team, Vienna, Austria) [22].

### Statistical analysis

Both complete and partial survey responses, including missing data, were retained for analysis. Descriptive statistics were used for continuous and categorical variables. Cell suppression techniques were applied, where appropriate, for reporting count variables with values between 1 and 10 for respondent characteristics. Free-text responses detailing specific PROMs used (by region) were collected and subsequently categorized to quantify the frequency of reported measures while addressing overlapping responses. Responses describing workflow patterns for PROM administration and data collection were summarized.

Linear regression was used to assess the bivariate relationship between self-reported use of PROMs at initial visits and subsequent/follow-up visits, hypothesizing a direct and positive relationship between self-reported PROM use for each visit type. To compare the central tendency of the distributions of responses, the Wilcoxon signed-rank test (non-parametric) was used given the paired nature of the data (derived from the same participants) and the non-normal distribution of the continuous values (0–100% scale) for PROM use for each type of visit. The test assesses whether there is a significant difference in the median values of the paired observations, with a single-side test performed for the hypothesis that reported PROM use at initial visits was significantly higher than at subsequent/follow-up visits. An a priori level of significance of α = 0.05 was used for all statistical analyses.

We also compared the self-reported frequency of use of PROMs by region of chief complaint. Survey items assess an ordinal self-reported frequency of PROM use (“Never”, “Several times per year”, “Several times per month”, “Several times per week”, “Several times per day”) across chief complaints common to chiropractic care – low back pain, neck pain, and all other chief complaints. A Kruskal-Wallis H Test was used to compare the nominal chief complaint variable with the ordinal PROM frequency variable; Dunn’s post-hoc tests with Bonferroni correction were performed to identify specific pairwise differences (low back pain vs. neck pain, low back pain vs. all other chief complaints, and neck pain vs. all other chief complaints). It was hypothesized that there would be no difference in PROM frequency between participants reporting low back pain and neck pain as their chief complaint, but that both groups would demonstrate significantly higher PROM frequency compared to those with other chief complaints. An a priori level of significance set at α = 0.05 was used for the initial Kruskal-Wallis test, with correction for multiple comparisons (i.e., corrected α=0.05/3) for Dunn’s post-hoc tests.

To describe respondent perceptions of PROM usefulness and burden during VHA chiropractic care, frequencies of Likert-style responses were used.

## Results

### Survey respondent characteristics

There were 365 invited participants with 189 providing full (*n* = 161) or partial (*n* = 28) response (51.8% overall response rate). Participant demographics are summarized in Table 1. Nearly all respondents reported being full-time VA employees, with most having between 1 and 5 years of VA service time.

**Table 1:** Respondent demographic characteristics (with cell suppression applied for count values 0 < *n* *≤* 10)

Respondent characteristic	Total
*Total responses*, *n* (%)	189
Complete responses	161 (85.2)
Partial responses	28 (14.8)
*Age*, *n *(%)	
20–29 years	> 14 (> 7.4)
30–39 years	61 (32.3)
40–49 years	52 (27.5)
50–59 years	34 (18.0)
Over 60 years	17 (9.0)
Prefer not to answer/missing	< 11 (< 5.8)
*Sex*,* n* (%)	
Female	65 (34.4)
Male	113 (59.8)
Prefer not to answer/missing	11 (5.8)
*Race*, *n *(%)	
White	151 (79.9)
Other race	14 (7.4)
Prefer not to answer/missing	24 (12.7)
*Ethnicity*, *n* (%)	
Hispanic or Latino	11 (5.8)
Not Hispanic or Latino	155 (82.0)
Prefer not to answer/Missing	23 (12.2)
*Military veteran status*, *n *(%)	
Yes	> 21 (11.1)
No	157 (83.1)
Prefer not to answer/missing	< 11 (< 5.8)
*VA position type*, *n* (%)	
VA employee	> 178 (> 94.2)
Other/Prefer not to answer/missing	< 11 (< 5.8)
*VA service time*, *n* (%)	
Less than 1 year	16 (8.5)
Between 1 and 5 years	101 (53.4)
Between 5 and 10 years	49 (25.9)
Greater than 10 years	> 12 (> 6.3)
Prefer not to answer/missing	< 11 (< 5.8)

### Patient reported outcome measure use

A complete listing of reported PROMs by area of chief complaint, their reported frequency, and the primary domain covered are presented in Online Appendix 1, Table 1. The PROMs most frequently listed by respondents assessed disability for both low back and neck pain: the Oswestry Disability Index (*n* = 75) and the Neck Disability Index (*n* = 63), respectively. Other commonly reported PROMs used to assess low back and neck pain addressed pain intensity, pain interference, disability, function, and risk stratification. Specific measures common to both low back and neck pain were the PEG-3 (*n* = 32 for low back pain; *n* = 30 for neck pain), Bournemouth Questionnaire (*n* = 31; *n* = 25), PROMIS measures (all versions/forms) (*n* = 31; *n* = 27), and the Keele STarT Back Screening Tool (*n* = 21; *n* = 9). The most reported PROMs used for all other chief complaints were the PEG-3 (*n* = 30) and PROMIS measures (all versions/forms) (*n* = 25). Additional common measures included function or disability questionnaires for specific regions, including the Disability of the Arm, Shoulder, and Hand Questionnaire (*n* = 21), the Lower Extremity Functional Scale (*n* = 18), and the Headache Disability Index (*n* = 14). Several reported PROMs were unable to be mapped to or did not represent widely accepted or validated instruments for low back pain, neck pain, and all other chief complaints.

The PROM administration workflow (Table 2) was most often completed by the chiropractor themselves (76.7%), with administration by other clinic staff (20.1%) or patient self-administration (7.9%) much less common. Write-in “Other” responses describing PROM administration most often described multiple people responsible for PROM administration/documentation across a broader clinic process – for example, distribution and scoring by a clinic staff member and documentation by the chiropractor.

**Table 2:** Patient reported outcome measure administration and data collection workflow characteristics from *n* = 189 responses

Item	Total (%)
*If you use patient reported outcome measures in your usual clinical practice, who is administers these measures (i.e., data collection) as part of your clinical workflow? (Select all that apply)*	
Myself	145 (76.7)
Another chiropractor in our clinic	10 (5.3)
Clinic staff (e.g., clinic nurse, MSA)	38 (20.1)
Patient self-administration	15 (7.9)
Other	4 (2.1)
Not applicable	16 (8.5)
*If you use patient reported outcome measures in your usual clinical practice, how are these data collected? (Select all that apply)*	
Using paper forms completed at the point of care	108 (57.1%)
Using paper forms provided to the patient to be completed asynchronously and returned during a visit	17 (9.0%)
Collected electronically at the point of care (e.g., using a computer, tablet, or mobile device during the visit)	18 (9.5%)
Collected electronically and asynchronously/remotely (such as using Annie, PETALS, or BHL Touch)	4 (2.1%)
Obtained through patient interview (e.g., the doctor asks the patient items on a given instrument)	57 (30.2%)
Other	3 (1.6%)
Not applicable	16 (8.5%)

Multiple selections were permitted for each workflow item, allowing for overlapping responses

The PROM data collection workflow (Table 2) most often involved use of paper forms completed at the point of care (51.7%) or through patient interview at the point of care (30.2%). Electronic data collection at the point of care (9.5%) or asynchronously (2.1%) or use of paper forms for asynchronous data collection (9.0%) were uncommon.

### Current use of patient reported outcome measures, by visit type

**Fig. 1:**
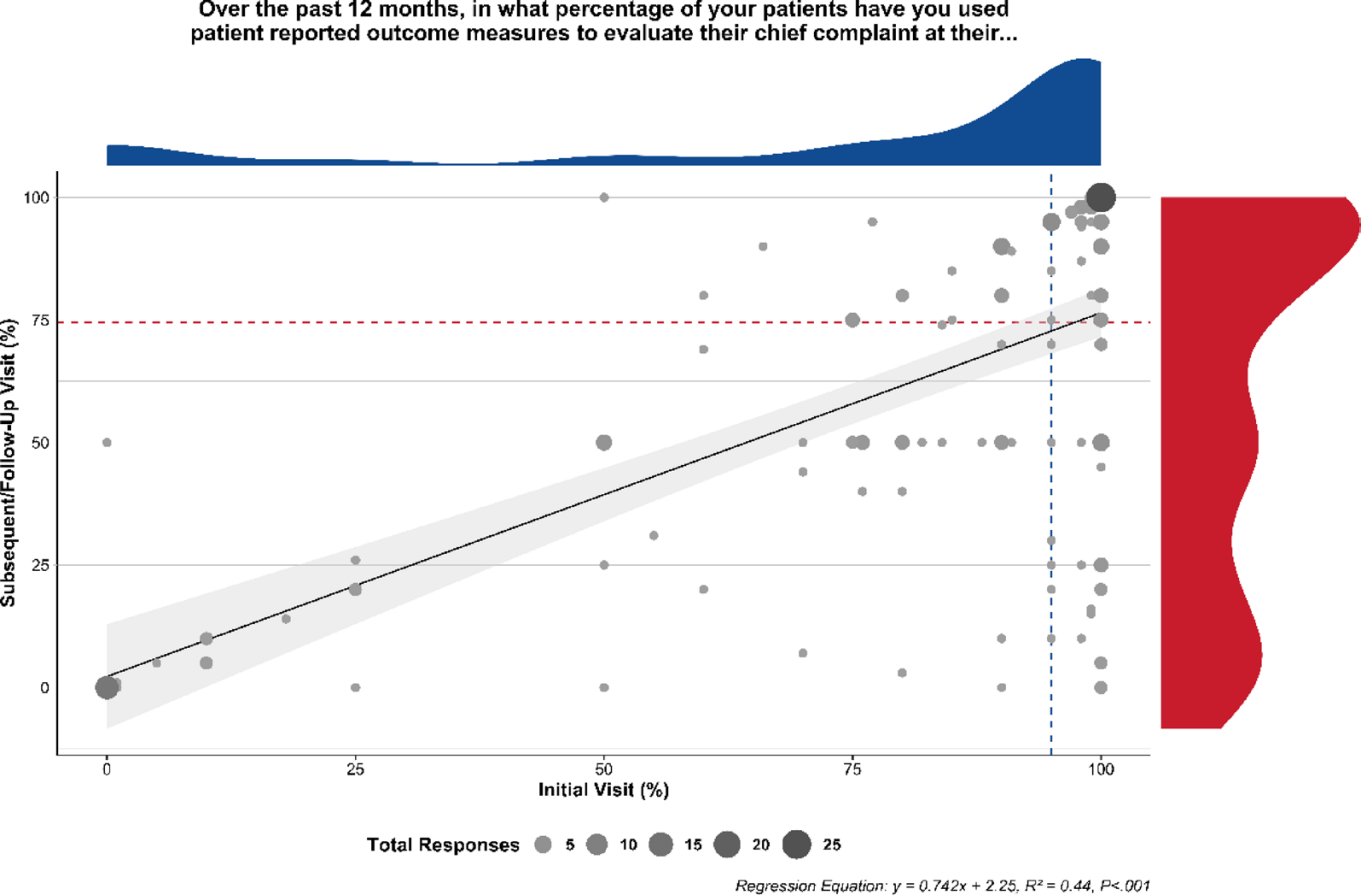
Percentage of patients for whom patient reported outcome measures were used, reported based on visit type. Marginal frequency distributions are presented, with dashed lines indicating the respective distribution medians. Regression is weighted for count of responses

The percentage of patients in the previous 12 months who used PROMs varied by initial versus subsequent/follow-up visit type (Fig. 1). Respondents reported a greater (Wilcoxon effect size *r* = 0.640, *p *< 0.001) percentage of patients over the past 12 months where they used PROMs to evaluate a chief complaint at initial visits (median = 95.0%, Q1–Q3: [75.0, 100.0]; mean = 78.1%, SD = 32.7, 95% CI [73.3, 83.0]; *n* = 12 missing) compared to subsequent/follow-up visits (median = 74.5%, Q1–Q3: [25.0, 95.0]; mean = 60.4%, SD = 36.7, 95% CI [54.9, 65.8]; *n* = 13 missing).

The bivariate relationship between self-reported PROM use at initial visits compared to subsequent/follow-up visits (Fig. 1) was positive and significant (β = 0.742, *p *< 0.001, 95% CI [0.617,0.868], R^2^ = 0.44). There were 121 respondents (64%) who indicated they used PROMs for more than 50% of their patients for both initial and subsequent/follow-up visits. Of the remaining respondents, 29 reported using PROMs at more than half of initial visits but less than half of follow-up visits, 25 reported using PROMs at less than half of both initial and follow-up visits, 1 reported using PROMs at less than half of initial visits but greater than half of follow-up visits, 1 reported using PROMs at greater than half of initial visits but did not provide a response for follow-up visits, and 12 did not provide responses for either initial or follow-up visits.

### Current use of patient reported outcome measures, by region of chief complaint

**Fig. 2:**
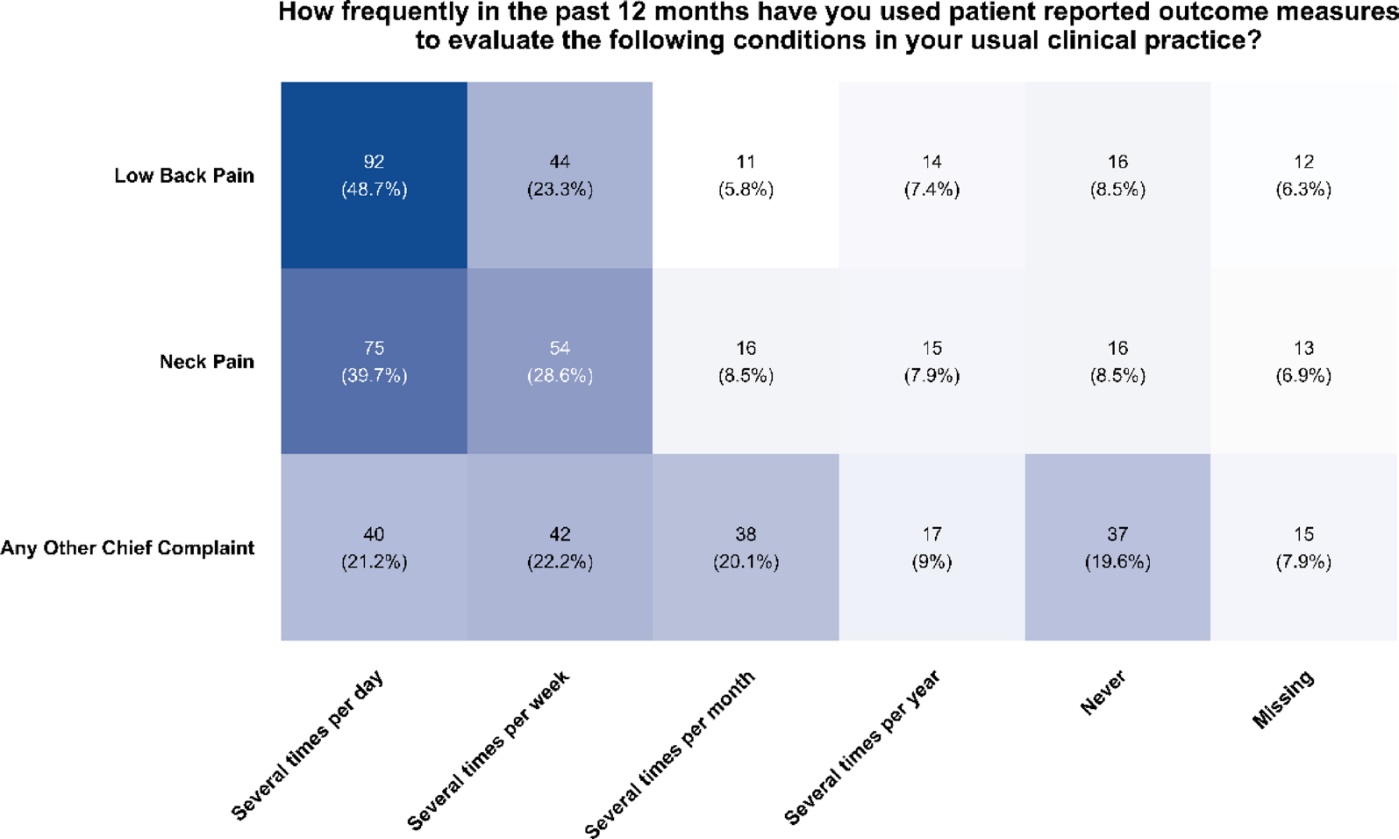
Frequency of use of patient reported outcome measures by region of chief complaint

Reported frequency of use of PROMs by region of chief complaint similarly varied across low back pain, neck pain, and other chief complaints (Fig. 2). Most respondents indicated using PROMs at least several times per week or per day for low back pain (72.0%) and neck pain (68.3%), with fewer than half indicating so for any other chief complaint (43.4%). Respondents indicating never using PROMs for any other chief complaint (19.6%) were more than double those indicating never using PROMs for low back pain (8.5%) or neck pain (8.5%).

The Kruskal-Wallis H test revealed a significant difference in PROM frequency across chief complaint categories (χ²=41.814, df = 2, *p *< 0.001). Post-hoc Dunn’s tests with Bonferroni correction did not find differences in PROM frequency observed between low back pain and neck pain chief complaints (rank-based effect size *r* = 0.055, *p *= 0.57). However, there was a difference found in reported PROM frequency for both low back pain (*r* = 0.258, *p *< 0.001) and neck pain (*r* = 0.203, *p *< 0.001) chief complaints compared to all other chief complaints.

### Usefulness and burden of patient-reported outcome measures collection

**Fig. 3:**
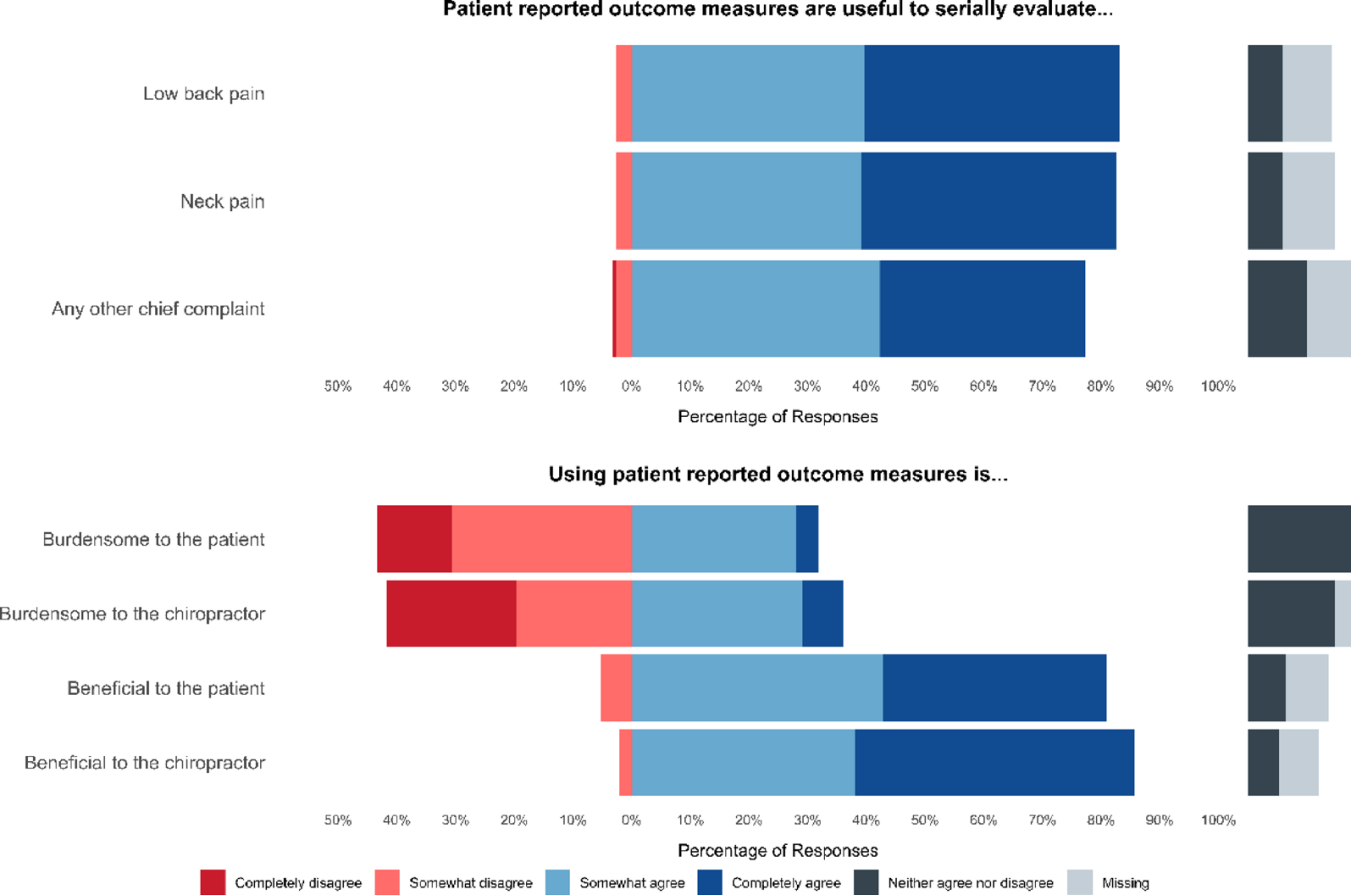
Chiropractor-perceived usefulness and burden of patient-reported outcome measures on patient and clinicians. Neutral and missing responses are offset

Respondents overwhelmingly indicated usefulness of PROMs to serially evaluate low back pain (83.1% “Completely agree” or “Somewhat agree” vs. 2.6% “Completely disagree” or “Somewhat disagree”), neck pain (82.6% vs. 2.6%), and any other chief complaint (77.2% vs. 3.1%) (Fig. 3 and Online Appendix 1, Tables 2 and 3). Respondents similarly indicated PROM use as beneficial to the patient (81.0% “Completely agree” or “Somewhat agree” vs. 5.3% “Completely disagree” or “Somewhat disagree”) and to the chiropractor (85.7% vs. 2.1%). There was greater balance in responses on PROM use being burdensome; 31.7% agreed and 43.4% disagreed that PROM use was burdensome to the patient, while 36.0% agreed and 41.8% disagreed that PROM use was burdensome to the chiropractor. There were more neutral responses on the perceptions of burden of PROMs (18.0% for patients, 14.8% for chiropractors) compared to their benefit (6.3% for patients, 5.3% for chiropractors).

## Discussion

In this cross-sectional survey of VHA Chiropractor perspectives on the use of PROMs in clinical practice, we identified several major themes including the specific measures used, administration workflow, and perceived utility. VHA chiropractors self-report a high commitment to PROM use across visit types and regions of chief complaint, with significantly greater use during initial consultation visits and for spinal complaints. This commitment is most often executed via a paper-based and provider-centric workflow. PROMs are overwhelmingly viewed as highly beneficial for individual patient management despite any perceived administrative burden.

### Patient reported outcome measure selection and use patterns

VHA chiropractors demonstrate strong alignment with current clinical practice guidelines for chronic spinal pain, with the most reported PROMs being validated, condition-specific disability measures (such as the Oswestry Disability Index and the Neck Disability Index). Many of the measures reported for low back pain are consistent with those reported in the broader chiropractic literature [23]. The simultaneous use of condition-specific tools alongside more flexible, global measures (e.g., the PEG-3 and PROMIS measures) could represent an approach to adopt a more holistic view of the patient’s health, considering multiple components of the biopsychosocial experience of pain [24]. Nonetheless, few individual respondents indicated a comprehensive list of PROMs used to address multiple domains.

Use patterns also varied significantly across different characteristics of chiropractic care. While the median reported use was high at initial visits (95.0%), this dropped with a strong effect during subsequent visits (74.5%), suggesting that PROM use may be framed more as an intake or baseline assessment rather than part of serial, measurement-based care critical for tracking treatment effect, informing care plan modification, and demonstrating ongoing care value. The variability in baseline and subsequent administration may also reflect the inconsistent serial use of PROMs potentially linked to challenges with administration frequency. This also highlights a potential area where automated digital health solutions may be leveraged to offload the cognitive load from clinicians for recalling measure administration timepoints while capitalizing on other benefits of technology-based communication [25].

While low back pain and neck pain saw high-frequency use (approximately 70% reported daily or weekly use), the frequency of use for all other chief complaints was significantly lower (43.4%). This suggests that while PROM use is an established part of the clinical culture for spinal pain, a consistent, whole-person measurement-based approach may not be uniformly applied across all musculoskeletal presentations within VHA chiropractic services. It’s also possible that this finding may be attributable to spinal pain complaints being far more common in VHA chiropractic care compared to all other chief complaints [13].

### Workflow and administrative burden

The efficiency and scalability of PROM collection are likely to be severely limited by the current VHA workflow. Our results indicate that the administration process is heavily provider-dependent, with the chiropractor primarily responsible for PROM completion in over three-quarters of the responses. Critically, data collection frequently relies on use of paper forms (51.7%) or patient interview at the point of care (30.2%). Given simple, electronic administration systems are facilitatory to PROM implementation [17], the provider-centric, paper-based approach is suspected to be a barrier to effective PROM implementation. Our findings point towards broader system design as a potential source of administrative strain and an opportunity for innovation and improvement in routine PROM collection.

Although providers overwhelmingly view PROMs as beneficial (81–86% agreement), a substantial minority (36.0%) agree that PROM use is burdensome to them, which we suspect may be attributable directly to current workflows. The low rates of electronic data collection (less than 12%) highlight an electronic information systems (i.e., EHR) integration gap. Electronic collection of PROMs demonstrates several benefits relative to traditional, paper-based methods including improved data quality, automated data processing, improved adherence and compliance, reduced administration time, and overall patient preference [26]. Importantly, these benefits should be weighed with patient privacy concerns, supporting infrastructure, or the potential to perpetuate a digital divide in some populations [26].

### Perceived utility and the value-based model

The high rates of self-reported use across visit types and clinical presentations, along with the substantial perceived benefit of PROMs for both the patient and the chiropractor, is a key finding. These combined findings highlight strong clinical buy-in among VHA chiropractors, despite a contradiction with previous findings of pragmatic use patterns in empirical analysis of VHA chiropractor documentation [16]. Our results suggest recognition of the perceived usefulness of PROMs in facilitating patient-provider communication and shared decision-making, consistent with reported facilitators of use [27]. However, the reliance on manual, point-of-care administration hinders a key systemic purpose of PROMs: to collect easily accessible, standardized data for outcome reporting and population health monitoring [4, 5]. To transition fully to a value-based care model, as increasingly emphasized by the VHA, clinical data must be easily transformed into system-level actionable insights. Our findings imply that, although the intent to measure is present, the method of measurement may be preventing VHA chiropractors from seamlessly contributing to nationally aggregated metrics. Addressing the workflow barriers is therefore mandatory to realize the full value proposition of measurement-based care for Veterans receiving chiropractic care.

### Strengths, limitations, and future directions

A primary strength of this study is the national scope, providing a representative sample of VHA chiropractors. While the moderate response rate may limit the generalizability of specific estimates, the national sample enhances the internal validity for system-wide reporting. Our study is the first to systematically capture the provider perspective on PROM selection and workflow in VHA chiropractic care, filling a critical gap left by previous VHA studies that focused primarily on the documentation of outcomes via natural language processing. However, we did not analyze how specific provider demographics may have influenced PROM utilization patterns, nor did we assess the impact of local institutional characteristics such as the use and workflows of PROM collection in the chiropractor’s department and/or broader overall facility. Additional limitations include potential self-report bias or desirability bias, in which respondents may have over-reported adherence to best practices (e.g., the high median initial visit use). We also acknowledge that the variation in PROM use identified across different types of visits may reflect a misinterpretation of survey items; specifically, respondents may have reported usage based on total subsequent visits rather than the percentage of unique patients followed over time or considered attrition of patients in their response. Furthermore, as the respondents were all VHA chiropractors working in the unique, integrated VHA environment, our findings are not necessarily generalizable to non-VHA and private practice settings. As a survey of VHA chiropractors, these findings also reflect only the self-reported provider perspective and may not account for the reality of practice patterns nor do they account for patient experiences or perceptions regarding PROM administration or burden. Incorporating the patient voice in future studies is an important component of meaningful community engagement necessary for successful implementation.

To transform the strong clinical buy-in reported by VHA chiropractors into systemic quality improvement, there is an opportunity to implement digital solutions to shift the administrative burden of PROM collection away from the provider and into asynchronous, automated patient data collection. The wide and varied collection of PROMs self-reported as used highlights a lack of standardization that may limit data aggregation for quality improvement and population health. This is particularly evident given both overlapping domains of assessment across different instruments and inclusion of assessments that are not widely accepted as validated PROMs. One example commonly reported in this study was the Keele Start Back Screening Tool—a self-report questionnaire that functions as a prognostic screening tool for stratified care rather than a traditional PROM and whose purpose contrasts with the stepped-care model that the VHA has favored for pain management. Establishing a standardized, core PROM set, mirroring initiatives like the Veterans Health Administration Pain Management, Opioid Safety, and Prescription Drug Monitoring Program (PMOP) Pain Measure Set [28], could improve data aggregation for Veteran population health monitoring in chiropractic care. Future research should also explore potential heterogeneity in PROM use patterns across provider subgroups, examining how factors such as years of practice and VHA experience, educational institution of clinical training, or local facility factors may drive meaningful differences in practices. Future research should also include experimental designs to test effectiveness and implementation of digital PROM collection workflows, along with qualitative assessments to understand the residual barriers to using PROMs in VHA chiropractic practice.

## Conclusion

VHA chiropractors report a strong commitment to measurement-based care, particularly for spinal pain presentations. Use patterns of PROMs varied by visit type and by clinical presentation. Respondents reported a broad array of validated, condition-specific measures typically used in practice and perceived this as highly beneficial to case management; yet, about a third of respondents felt that PROM use was burdensome to the chiropractor and/or the patient. Since existing workflows often relied on provider collection via manual and paper-based methods, our findings suggest there is likely an opportunity to explore how electronic PROM collection may facilitate further uptake at the patient-provider interface. Such efforts could also bolster more effective data integration, enhanced data quality, and use for population health monitoring. To remain a viable solution, the implementation of such digital tools should balance these benefits against potential drawbacks. Addressing systemic and workflow barriers, while accounting for both the advantages and challenges of modernized digital data collection methods, is a key step to effectively implementing PROMs in VHA chiropractic practices and fully realize the value of measurement-based care for Veteran patients.

## Supplementary Information

Below is the link to the electronic supplementary material.

Supplementary Material 1

## Data Availability

The complete survey instrument and de-identified, minimally necessary datasets have been uploaded to the Open Science Framework (OSF) and are publicly available at 10.17605/OSF.IO/N8RUY.
