# Supplementary material for: Chiropractor perspectives on the use of patient reported outcome measures: a survey of Veterans Health Administration chiropractors
Source: Chiropr Man Therap. 2026 Mar 18;34:16. doi: 10.1186/s12998-026-00631-5 (PMC13112770; doi:10.1186/s12998-026-00631-5)
Supplement: Supplementary file 1 — Supplementary Material 1 [file 12998_2026_631_MOESM1_ESM.docx]

**Appendix 1, Table 1.** Patient reported outcome measures self-reported as used by respondents for low back pain (LBP), neck pain (NP), and all other chief complaints. Measures in the table are for those reported by 5 or more respondents, with other measures reported less than 5 times summarized below the table. Note infrequently reported measures are reported verbatim and may not reflect validated patient reported outcome measures.

| **Measure** | **Domain** | **Count (N)** | | |
| --- | --- | --- | --- | --- |
|  |  | **LBP** | **NP** | **Other** |
| Oswestry Disability Index | Physical function/disability | 75 | 11 | - |
| *PEG-3* | Pain intensity and interference | 32 | 30 | 30 |
| *Bournemouth Questionnaire* | Multiple domains (pain intensity, function/disability, affective components) | 31 | 25 | 5 |
| *PROMIS Measures (all version/forms)* | Multiple domains (including function, interference) | 31 | 27 | 25 |
| *Keele STarT Back Screening Tool* | Risk stratification for prognosis | 21 | 9 | 6 |
| *Functional Rating Index* | Physical function/disability | 17 | 16 | 13 |
| *Visual Analog Scale* | Pain intensity | 15 | 14 | 12 |
| *Defense and Veterans Pain Rating Scale (including supplemental items)* | Pain intensity (supplemental items on function) | 10 | 10 | 11 |
| *Numeric Rating Scale* | Pain intensity | 8 | 7 | 5 |
| *Patient Specific Functional Scale* | Physical function | 6 | 6 | 6 |
| *Roland Morris Disability Questionnaire* | Physical function/disability | 6 | - | - |
| *Global Perceived Improvement* | Overall health status (treatment response) | 5 | 5 | - |
| *Neck Disability Index* | Physical function/disability | - | 63 | - |
| *Disability of the Arm, Shoulder, and Hand Questionnaire* | Physical function/disability | - | - | 21 |
| *Lower Extremity Functional Scale* | Physical function/disability | - | - | 18 |
| *Headache Disability Index* | Disability/impact | - | - | 14 |
| *QuickDASH* | Physical function/disability | - | - | 8 |
| *Upper Extremity Functional Index* | Physical function/disability | - | - | 5 |

**Measures reported <5 times for Low Back Pain:** ‘*Activities of Daily Living', 'Global Rating of Change', 'Patient Global Impression of Change', 'Quadruple Visual Analog Scale', 'Range of Motion', 'UCLA Loneliness Scale', 'Yellow Flag Questionnaire', 'Analog Pain Scale', 'Back Bothersome', 'Brief Pain Inventory', 'Graded Chronic Pain Scale', 'HSI', 'NDI', 'Neck and Back Questionnaire', 'Pain FID', 'Pain Index', 'Pain Meds', 'Physical Function and Interference Worksheet', 'SSI', 'Tampa Kinesiophobia Scale', 'UAB LifeSpace', 'VDS’*

**Measures reported <5 times for Neck Pain:** *‘Activities of Daily Living', 'Neck Pain Index', 'Patient Global Impression of Change', 'Quadruple Visual Analog Scale', 'Range of Motion', 'UCLA Loneliness Scale', 'Global Rating of Change', 'Vernon Mior', 'Analog Pain Scale', 'Headache Disability Index', 'Neck Bothersome Index', 'Pain Medications', 'VDS', 'Yellow Flag Questionnaire', 'Brief Pain Inventory', 'Graded Chronic Pain Scale', 'NPS', 'Pain FID', 'Physical Function and Interference Worksheet’*

**Measures reported <5 times All Other Chief Complaints:** ‘Oswestry', 'Shoulder Pain and Disability Index', 'Patient Global Impression of Change', 'Tampa Kinesiophobia Scale', 'UCLA Loneliness Scale', 'Activities of Daily Living', 'Back Bothersome Index', 'Cozean's Pelvic Dysfunction Screening Protocol', 'Global Perceived Improvement', 'Global Rating of Change', 'Yellow Flag Questionnaire', 'Range of Motion', 'Analog Pain Scale', 'Ankle Disability', 'Brief Pain Inventory', 'Brief Headache Screen', 'Fear Avoidance Beliefs Questionnaire', 'Functional Measures/Statements of Activity', 'Graded Chronic Pain Scale', 'Headache Impact Test-6', 'HIT-6', 'ID Migraine Screen', 'MCPMSS', 'NDI', 'Pain Drawing', 'Pain FID', 'Pain Medications', 'Physical Function and Interference Worksheet', 'QVAS', 'SF-36', 'Simple Shoulder ', 'UE Function', 'VDS', 'Whole Health', 'WOMAC’

**Appendix 1, Table 2.** Respondent perceptions of PROM usefulness by area of chief complaint (n=189 respondents).

| **Patient reported outcome measures are useful to serially evaluate…** | |
| --- | --- |
| **Low back pain** |  |
| *Completely disagree* | 0 (0%) |
| *Somewhat disagree* | 5 (2.6%) |
| *Neither agree nor disagree* | 11 (5.8%) |
| *Somewhat agree* | 75 (39.7%) |
| *Completely agree* | 82 (43.4%) |
| *Missing* | 16 (8.5%) |
| **Neck pain** |  |
| *Completely disagree* | 0 (0%) |
| *Somewhat disagree* | 5 (2.6%) |
| *Neither agree nor disagree* | 11 (5.8%) |
| *Somewhat agree* | 74 (39.2%) |
| *Completely agree* | 82 (43.4%) |
| *Missing* | 17 (9.0%) |
| **Any other chief complaint** |  |
| *Completely disagree* | 1 (0.5%) |
| *Somewhat disagree* | 5 (2.6%) |
| *Neither agree nor disagree* | 19 (10.1%) |
| *Somewhat agree* | 80 (42.3%) |
| *Completely agree* | 66 (34.9%) |
| *Missing* | 18 (9.5%) |

**Appendix 1, Table 3.** Respondent perceptions of PROM burden and benefit to the patient and chiropractor (n=189 respondents).

| **Using patient reported outcome measures is…** | |
| --- | --- |
| **Burdensome to the patient** |  |
| *Completely disagree* | 24 (12.7%) |
| *Somewhat disagree* | 58 (30.7%) |
| *Neither agree nor disagree* | 34 (18.0%) |
| *Somewhat agree* | 53 (28.0%) |
| *Completely agree* | 7 (3.7%) |
| *Missing* | 13 (6.9%) |
| **Burdensome to the chiropractor** |  |
| *Completely disagree* | 42 (22.2%) |
| *Somewhat disagree* | 37 (19.6%) |
| *Neither agree nor disagree* | 28 (14.8%) |
| *Somewhat agree* | 55 (29.1%) |
| *Completely agree* | 13 (6.9%) |
| *Missing* | 14 (7.4%) |
| **Beneficial to the patient** |  |
| *Completely disagree* | 0 (0%) |
| *Somewhat disagree* | 10 (5.3%) |
| *Neither agree nor disagree* | 12 (6.3%) |
| *Somewhat agree* | 81 (42.9%) |
| *Completely agree* | 72 (38.1%) |
| *Missing* | 14 (7.4%) |
| **Beneficial to the chiropractor** |  |
| *Completely disagree* | 0 (0%) |
| *Somewhat disagree* | 4 (2.1%) |
| *Neither agree nor disagree* | 10 (5.3%) |
| *Somewhat agree* | 72 (38.1%) |
| *Completely agree* | 90 (47.6%) |
| *Missing* | 13 (6.9%) |
